# The Impact of Job Insecurity on Knowledge-Hiding Behavior: The Mediating Role of Organizational Identification and the Buffering Role of Coaching Leadership

**DOI:** 10.3390/ijerph192316017

**Published:** 2022-11-30

**Authors:** Jeeyoon Jeong, Byung-Jik Kim, Min-Jik Kim

**Affiliations:** 1 Korea University Business School, Korea University, Seoul 02841, Republic of Korea; 2 Department of Psychology, Yonsei University, Seoul 06695, Republic of Korea; 3 College of Business Administration, University of Ulsan, Ulsan 44610, Republic of Korea; 4 School of Industrial Management, Korea University of Technology and Education, 1600, Chungjeol-ro, Cheonan 31253, Republic of Korea

**Keywords:** job insecurity, organizational identification, knowledge-hiding behavior, coaching leadership, moderated mediation

## Abstract

As the global economic situation deteriorates due to the prolonged COVID-19 pandemic, the business environment is plagued by uncertainty and risk. To address this, many organizations have sought to optimize efficiency, especially by downsizing and restructuring, to reduce costs. This causes anxiety among employees, who worry about whether they will be fired. We hypothesize that such job insecurity increases knowledge-hiding behavior by employees, and we investigate the mechanism underlying such a negative effect. In addition, we attempt to capture the boundary conditions of how to reduce the adverse effects of job insecurity, focusing on the role of coaching leadership. Using three-wave time-lagged cohort-study data from 346 Korean workers, we empirically found that employees who perceive job insecurity are less likely to feel organizational identification, leading to increased knowledge-hiding behavior. This study also demonstrated that coaching leadership operates as a boundary condition which buffers the negative influence of job insecurity on organizational identification. Theoretical and practical implications are discussed.

## 1. Introduction

The outbreak of the COVID-19 pandemic has caused difficulty around the world. Many employees around the globe lost their jobs due to the COVID-19 pandemic [1,2]. Likewise, rapid organizational changes such as the emergence of artificial intelligence (AI) and robot process automation (RPA) have increased employee job-insecurity, which corresponds to subjective perceptions of the threat of employment instability [3]. Thus, organizational managers need to not only understand how employee uncertainty about employment has negative effects on employees and the organization, but also how to cope with negative impacts [4,5,6]. Previous studies examined the influences of job insecurity on perceptions, attitudes, and behaviors of employees, such as organizational commitment, organizational identification, job satisfaction, meaning of work, organizational trust, and organizational citizenship behavior, as well as job stress, emotional exhaustion, turnover intention, and organizational deviance [7,8,9,10,11].

Although extant studies have dealt with various effects of job insecurity on employees, we believe that some research gaps exist that have been underexplored. First, studies have relatively underexplored employees’ “knowledge-related” behaviors [11,12]. Previous research has mainly demonstrated that job insecurity is closely associated with employee behaviors such as organizational citizenship behavior, innovative behavior, voice behavior, counterproductive work behavior, and safety behavior [12,13,14,15,16]. These are critical organizational outcomes. However, knowledge is the fundamental and essential source of service and product innovation, as well as the generator of added value, eventually determining the competitive advantage of organizations [17,18,19]. We expect that job insecurity would cause the deterioration of the employees’ perceptions and attitudes toward their organization. The decreased quality of employees’ attitudes towards the organization are then likely to increase their harmful behaviors, such as knowledge-hiding behavior based on the social exchange perspective [11,12,13,17,19]. These are the reasons why we suggest that job insecurity may increase their knowledge-hiding behavior. Therefore, investigating the influence of job insecurity on the knowledge-related behavior of employees is highly recommended.

Second, to the best of our knowledge, few studies of job insecurity have investigated the mediators and moderators of the relationship between job insecurity and knowledge-related behaviors [11,12]. As mediators and moderators may reveal why and when the associations between job insecurity and knowledge-related behaviors are established in an organization, delving into the underlying processes and contextual variables is critical. Therefore, studies of the mediators and moderators of job insecurity-knowledge-related behaviors are needed.

Third, and most importantly, extant research on job insecurity has underexplored the role of leadership in an organization [11,12]. Although there have been studies of boundary conditions that buffer the negative effects of job insecurity, most have focused on individual-level variables such as self-esteem, the internal locus of control, proactive personality, psychological capital, resilience, and emotional intelligence [20,21,22,23,24,25], or macro-level contextual factors such as labor-market insecurity, social safety networks, and macro-economic conditions [11,12]. Considering that leaders play critical roles in building subordinates’ perceptions, attitudes, and behaviors by assigning tasks, evaluating performance, and establishing implicit norms or rules in an organization [26,27,28], investigating the moderating role of leadership is requested.

To fill the existing research gaps, in this study we focus on the intermediating mechanisms and contextual factors in the relationship between job insecurity and knowledge-hiding behavior. To be specific, we suggest that not only the level of employee organizational-identification may mediate the link between job insecurity and knowledge-hiding behavior, but also coaching leadership would positively moderate the job insecurity-organizational-identification link. Knowledge-hiding behavior refers to the act of intentionally withholding or concealing knowledge from another employee who requested it [29]. Organizational identification (OI) refers to the degree to which an employee perceives a sense of belonging, unity, or oneness with an organization [30,31,32,33,34,35]. Based on previous studies [11,36,37,38], we suggest that job insecurity decreases employee organizational-identification. In turn, decreased organizational-identification may increase harmful behaviors towards the organization, such as knowledge-hiding behavior, organizational deviance, and interpersonal deviance [39,40,41,42].

This mediation structure can be explained by the psychological-contract-breach perspective [43,44], which suggests that an employee’s perception on the breach of the psychological contract would cause the deterioration of the quality of his or her attitudes and behavior in an organization.

Furthermore, as various contextual factors affect employee responses to job insecurity [23,28,45], we suggest that coaching leadership positively moderates the relationship between job insecurity and organizational identification by buffering the harmful effects of job insecurity [46,47,48]. Among various kinds of leadership styles, we selected coaching leadership, because employees who feel a sense of job insecurity would feel psychological difficulties such as anxiety, depression, and anger, which require being cared for, soothed, supported, and guided by an authority figure such as a leader [3,6,9].

**Figure 1:**
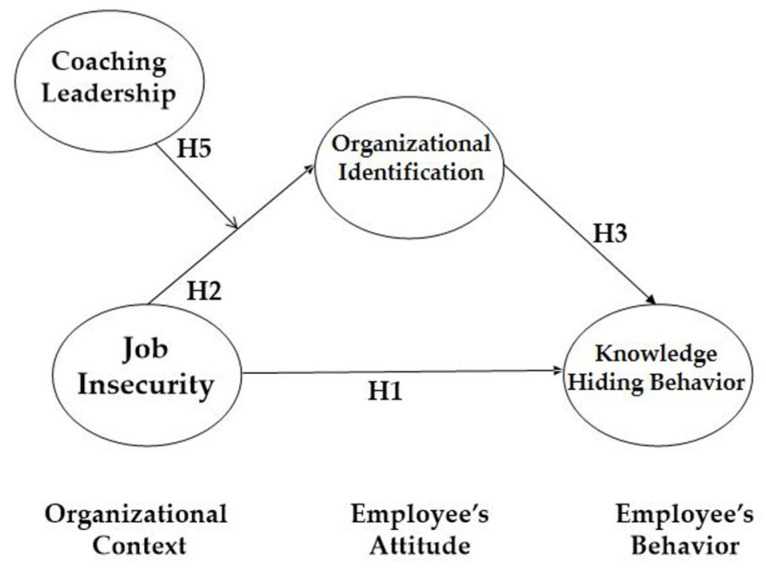
Theoretical model (T1, T2, and T3 mean time point 1, 2, and 3, respectively).

In summary, the aims of this study are to delve into the influence of job insecurity on knowledge-hiding behavior through the mediating effect of organizational identification. In addition, we suggest that coaching leadership positively moderates the relationship between job insecurity and organizational identification. To empirically test these hypotheses, we established a moderated mediation model with structural equation modeling (SEM). We believe that this paper contributes to the job-insecurity literature, as follows: first, we investigate the influence of job insecurity on knowledge-related behavior, specifically knowledge-hiding behavior. Second, we delve into the mediators in the relationship between job insecurity and knowledge-hiding behavior. The degree of employee identification with the organization functions as the underlying mechanism of the link. Third, we examine the boundary conditions or contextual factors (moderators) that play buffering roles, by positively moderating the job-insecurity–organizational-identification link. Lastly, we collected data over three time-periods to decrease the harmful impacts of common-method bias in the cross-sectional research design. We summarize our proposed relationship as follows (please see Figure 1).

## 2. Theories and Hypotheses

### 2.1. Job Insecurity and Knowledge-Hiding Behavior

In this paper, we suggest that job insecurity would increase the degree of employees’ knowledge-hiding behavior [49]. According to the results of many previous studies [7,8,9,10,11,17], job insecurity causes the deterioration of the quality of employees’ perceptions and attitudes toward their organization (e.g., organizational identification). Therefore, this diminished degree of employees’ attitudes towards the organization is likely to increase their harmful behaviors toward the organization, including knowledge-hiding behavior based on the social exchange perspective [11,12,13,17,19,49]. Based on these arguments, we suggest that job insecurity may increase employees’ knowledge-hiding behavior.

**Hypothesis 1.**  *Job insecurity may increase the degree of knowledge-hiding behavior*.

### 2.2. Job Insecurity and Organizational Identification

In the current research, we hypothesize that job insecurity decreases employee organizational-identification. This concept not only determines organizational success and failure, but also promotes organizational cooperation, welfare, job commitment, and work performance [31,32,33,34,35]. Organizational identification is a prominent form of social identity, which consists of the components of individual identity and self-concept. We suggest several reasons why job insecurity undermines organizational identification. First, individuals tend to meet their belongingness needs by forming group identities. Job insecurity presents an unpleasant, negative organizational-experience that undermines employees’ feelings of belonging. Job insecurity implies anxiety and the danger that employees may be fired at any time [6,11,12]. As a result, employees feel worthless as members of the organization and they feel that their desire to belong is difficult to realize. Therefore, job insecurity weakens employee identification with the organization, as it obstructs their need for belonging. 

Second, the more employees feel appreciated and respected within the organization, the more they identify with the organization. Job insecurity weakens employee senses of well-being and value within the organization, resulting in low organizational-identification. When an organization recognizes the value of its employees and cares about their safety, employees feel like organizational insiders, which promotes identification with the organization. On the other hand, when an organization does not care about employee safety and well-being, employees feel worthless, and that they are outsiders. Accordingly, employees who feel job insecurity lower their identification with the organization, as they feel they are not receiving support, respect, or care from the organization. Taken together, we propose the following hypothesis.

**Hypothesis 2.**  *Job insecurity may decrease the degree of organizational identification*.

### 2.3. Organizational Identification and Knowledge-Hiding Behavior

We suggest that organizational identification decreases knowledge-hiding behavior. Knowledge-hiding behavior refers to the act of intentionally withholding or concealing knowledge from another employee who requested it [29]. Employees with high organizational identification feel psychologically connected to the organization, have shared values with the organization, and feel a sense of belonging within the organization [50,51]. Such high organizational-identification not only inspires employees to have positive attitudes and behaviors toward the organization, but also to do their best for the organization [52,53]. Extant research revealed that organizational identification has positive relationships with job satisfaction, job engagement, in-role performance, and organizational citizenship behavior [54]. Employees who strongly identify with the organization follow the standards and norms of the organization, internalize the organization’s goals as their own goals, and treat other organizational members positively [55]. In addition, although existing studies have mainly revealed that organizational identification is related to positive behaviors such as organizational citizenship, few studies have examined the relationship between organizational identification and negative outcomes such as absenteeism, turnover intention, and counterproductive work-behavior [28,56,57,58,59]. 

Based on these studies, we can expect that organizational identification is negatively related to knowledge-hiding behavior, since the behavior is a typical type of counterproductive work-behavior that intentionally hinders and prevents organizational goals and success. Such behavior prevents firms from effectively coping with radical environments in achieving successful service and product innovation, reduces employee creativity, and hinders organizational innovativeness [17,18,19]. Therefore, employees with high organizational identification would try to avoid knowledge-hiding behavior to contribute to achieving his or her organization’s success. 

To be specific, the influence of organizational identification on knowledge-hiding behavior can be explained by conservation of resources (COR) theory. According to this perspective, an employee is likely to restore his or her valuable resources from being lost, when he or she experiences negative perceptions or attitudes [60]. Job insecurity would increase the employee’s negative attitudes toward the organization, decreasing the level of organizational identification. Then, in this situation, the employee would try to do his or her best to conserve his or her valuable resource such as ‘knowledge’ in an organization [17,60]. In other words, an employee who has a low level of organizational identification is likely to hide his or her knowledge to restore his or her resources in an organization. Strictly speaking, the knowledge-hiding behavior can be defined as intentional behavior toward the other employees, not the organizations. However, we consider that the employees who conduct the hiding behavior are likely to perceive that their hiding behavior would be eventually harmful to the organization. This is the reason why this paper connects organizational identification with knowledge-hiding behavior.

Thus, we suggest the following hypothesis.

**Hypothesis 3.**  *Organizational identification may decrease the degree of knowledge-hiding behavior*.

### 2.4. Mediating Role of Organizational Identification between Job Insecurity and Knowledge-Hiding Behavior

By integrating the above hypotheses, we propose that organizational identification mediates the relationship between job insecurity and knowledge-hiding behavior. Although there are no existing studies that address this using a single research model, we hypothesize that job insecurity reduces organizational identification, and reduced organizational identification increases knowledge-hiding behavior.

To more precisely explore this mediating effect, we attempt to apply the psychological contract breach perspective [43,44] to our research context. This theory suggests that an employee’s perception of the breach of the psychological contract would deteriorate the quality of his or her attitudes and behavior in an organization. Considering that job insecurity felt by an employee is likely to increase his or her perception of psychological contract breach, we can expect that job insecurity may have a significant impact on employee attitudes (i.e., organizational identification) and behaviors (i.e., knowledge-hiding behavior). 

In addition, the mediating effect of organizational identification between job insecurity and knowledge-hiding behavior can be described by conservation of resources (COR) theory. This theory suggests that an employee’s negative attitudes cause him or her to restore his or her precious resources in an organization [60,61]. Job insecurity would decrease the degree of employee’ organizational identification, because job insecurity increases the employee’s negative attitudes toward the organization. In this situation, the employee with decreased level of organizational identification is likely to attempt to restore his or her valuable resource such as ‘knowledge’ [17,60]. In other words, an employee who has a low level of organizational identification is likely to hide his or her knowledge to conserve his or her resources in an organization

Therefore, applying the rationale of the perspective, we suggest that organizational identification plays a mediating role in the relationship between job insecurity and knowledge-hiding behavior. Existing studies find that job insecurity decreases organizational identification [60]. We therefore hypothesize that organizational identification is negatively related to knowledge-hiding behavior. Thus, we propose that employee organizational-identification serves as an underlying behavioral mechanism in the relationship between job insecurity and knowledge-hiding behavior. Therefore, we propose the following hypothesis:

**Hypothesis 4.**  *Organizational identification mediates the relationship between job insecurity and knowledge-hiding behavior*.

### 2.5. Moderating Effect of Coaching Leadership on the Job Insecurity–Organizational Identification Link

Based on the preceding logic, it is certain that job insecurity undermines organizational identification. However, we assume that this relationship will not apply to all employees. This is because in a real organization, various factors may moderate the relationship between job insecurity and organizational identification. In other words, although the job insecurity that an employee feels within an organization reduces their organizational identification, it can be mitigated or strengthened according to individual characteristics (personality, gender, age) or situational factors (leadership or organizational climate) [11,45]. In particular, we suggest that coaching leadership alleviates the negative relationship between job insecurity and organizational identification. Since leadership represents the organization, it has a significant influence on the emotions, attitudes, and behaviors of subordinates [26,27]. In particular, we suggest that coaching leadership buffers undermined organizational-identification among subordinates, due to job insecurity. 

Specifically, we hypothesize that the higher the level of coaching leadership, the less the negative impact of job insecurity on subordinates’ organizational identification. Coaching leadership can be defined as leadership behavior that helps subordinates effectively solve and cope with problems, difficulties, or conflicts within an organization, thereby improving their performance and helping them fully realize their potential and growth [47,48]. Through coaching leadership, subordinates learn about their capabilities, possibilities, and strengths within the organization, and are motivated to further develop them for organizational performance. According to Heslin and his colleagues [62], coaching leadership consists of three components: (1) guidance, (2) facilitation, and (3) inspiration. First, guidance refers to providing constructive and positive feedback to subordinates on specific organizational expectations and goals, and how to achieve them. Facilitation means helping subordinates analyze and explore how to solve job-related problems and improve their performance on their own. Inspiration involves helping subordinates recognize their potential and value, motivating them to achieve better performance. Extant studies have also found that coaching leadership has positive effects on psychological well-being, job satisfaction, job performance, and organizational citizenship behavior [47,63,64].

More specially, we propose that coaching leadership functions to mitigate the detrimental effects of job insecurity on employee organizational-identification. Coaching leadership guides, facilitates, and inspires subordinates, thereby developing their potential, enhancing their capabilities, and achieving high self-efficacy [47,48]. As coaching leadership increases, employees feel more respect and support from their leader [47]. This causes subordinates to have positive self-concepts within the organization, and they feel respected and recognized by the organization. Therefore, even when employees feel high levels of job insecurity, coaching leadership reduces the negative effect by helping them address and cope with difficulties, anxiety, and fears, leading them to develop their growth and self-concepts within an organization. As a result, reduced organizational identification due to job insecurity is alleviated. In other words, coaching leadership plays a buffering role in the relationship between job insecurity and organizational identification. 

In contrast, low levels of coaching leadership make it difficult for subordinates to solve problems that arise within the organization, which in turn makes them feel less respected and recognized by the organization [47]. Thus, low coaching-leadership will make employees who feel insecure about employment perceive that they are not treated properly and do not receive support from the organization, decreasing their identification with the organization. Therefore, the negative effect of job insecurity on organizational identification is worsened in the presence of low coaching-leadership. We propose the following hypothesis.

**Hypothesis 5.**  *The negative relationship between job insecurity and organizational identification is moderated by coaching leadership. Specifically, when coaching leadership is high, the negative relationship between job insecurity and organizational identification will be alleviated, compared to when it is low*.

## 3. Method

### 3.1. Participants and Procedure

We used the online-survey system of a top-tier research company including approximately 3,300,000 research panelists, the largest research panel available in Korea. For the current study, we collected data from employees and their immediate supervisors who are currently working at South Korean firms. They were recruited through an online-survey company. The participants reported their occupation status when they registered for the online membership, via a user authentication system (e.g., cellular phone number or email address). Such online-survey systems are known as a reliable method for accessing various samples. 

The research is a cohort-design study, which is a particular form of longitudinal study, sampling a cohort by performing a cross-section at intervals, three times. In other words, this paper gathered data during three different time-periods. The panelists were randomly selected, to reduce the possibility of sampling bias. The online system’s operating functions allowed us to track who responded to our survey, confirming that participants from time-point one to time-point three were the same. Our survey system was open for two or three days each at each time-point, to provide enough time for participants to respond. When the system was open, participants could access it whenever they wanted. The company monitored the integrity of data by using traps for geo-IP violators and timestamps to flag efficient responding, which restricted participants from logging onto the survey site and filling out the surveys multiple times.

The experts in the research firm directly contacted the participants to ask for permission for participation in our survey, assuring them not only that their participation would be voluntary but that also their responses would be confidential and be used for only research purpose. In addition, the company reported and obtained both informed consent and compliance with ethical requirements from those who agreed with to participate. The company provided the participants with a reward for their participation in the form of cash (USD 8).

During the three time-periods, 512, 431, and 354 employees participated in surveys, respectively. By relying on the suggestions of previous works [11,12], the time-periods were approximately five or six weeks long. We then deleted missing data from the raw data. Finally, we utilized data from 346 employees and 346 supervisors for analysis (response rate: 67.58%). The 346 supervisors participated in our survey through the online system of the research company. 

To determine the sample size, we utilized various suggestions from previous research. First, we checked whether our sample size was appropriate through calculating the minimum sample-size using G*Power version 3.1.9.7. A power analysis with the program demonstrated that a sample size of 348 provided sufficient power (≥0.80) to detect a medium effect with an alpha level of *p* = 0.05 [65]. The characteristics of the participants are shown in Table 1.

### 3.2. Measures

The survey measured distinct variables in our research model at each time-point. At time point one, the respondents were asked about levels of job insecurity and coaching leadership. At time point two, we measured employee organizational-identification. At time point three, we assessed participants’ knowledge-hiding behavior, by surveying his or her immediate supervisor. These variables were assessed through multi-item scales on a five-point Likert scale (1 = strongly disagree, 5 = strongly agree). We used Cronbach alpha values to determine the internal consistency of each variable.

#### 3.2.1. Job Insecurity (Time Point One, Collected from Employees)

We used five items to measure the job-insecurity scale, which consists of ten items [66]. The reason why we shortened the full items is that the five items were validated by previous empirical research which was conducted in the context of South Korea [28]. Sample items are, “If my current organization were facing economic problems, my job would be the first to go”, “I will not be able to keep my present job as long as I wish”, and “My job is not a secure one”. The Cronbach’s alpha value was 0.90.

#### 3.2.2. Coaching Leadership (Time Point One, Collected from Employees)

To measure the degree of coaching leadership, we utilized twelve items from previous studies on coaching leadership [47,48]. The reason why we shortened the full items is that the twelve items were validated by existing empirical research which was conducted in the context of South Korea [64]. Sample items are, “My leader believes in my potential for growth’, and ‘My leader asks questions that make me reflect on my thoughts and perspectives’. The Cronbach’s alpha value was 0.94.

#### 3.2.3. Organizational Identification (Time Point Two, Collected from Employees)

To measure employees’ organizational identification, we utilized five items from Mael and Ashforth’s scale [50]. The reason why we shortened the full items is that the five items were validated by extant empirical research which was conducted in the context of South Korea [56]. Sample items are, “When someone criticizes my organization, it feels like a personal insult”, and “My organization’s successes are my successes”. The Cronbach’s alpha value was 0.81.

#### 3.2.4. Knowledge-Hiding Behavior (Time Point Three, Collected from Employees’ Immediate Supervisors)

The degree of employee knowledge-hiding behavior was measured using five items of the knowledge-hiding behavior scale, which consists of eleven items [67,68]. Each employee’s immediate supervisor evaluated the level of his or her knowledge-hiding behavior. The reason why we shortened the full items is that the five items were validated by extant empirical research, which w conducted in the context of South Korea [69]. A sample item is, “This employee pretended that he or she couldn’t find the information that his or her colleagues wanted”, and “This employee gives colleagues a little bit of assistance, but didn’t help them to the extent they wanted”. The Cronbach’s alpha value was 0.81.

#### 3.2.5. Control Variables

Based on previous studies [67,68,69], the dependent variable of this research, knowledge-hiding behavior, was controlled by employee factors such as tenure, gender, position, and education. The control variables were collected at time point two.

### 3.3. Statistical Analysis

Frequency analysis was performed to assess the participants’ demographic features. We conducted a Pearson correlation analysis with SPSS 26 to compute the relationships among our research variables. Then, following Anderson and Gerbing [70], we took a two-step approach that consists of (1) measurement, and (2) the structural model. To test the validity of the measurement model, we performed a confirmatory factor analysis (CFA). Next, based on SEM, we performed a moderated-mediation-model analysis with the maximum likelihood (ML) estimator using AMOS 23, to test the structural model. 

To test whether various model fit-indexes are acceptable, we utilized a variety of goodness-of-fit indices, including the comparative fit index (CFI), the Tucker–Lewis index (TLI), and the root-mean-square error of approximation (RMSEA). Extant research has reported that CFI and TLI values greater than 0.90 and RMSEA values of less than 0.06 are appropriate [71]. Finally, to check whether our mediation hypothesis was supported, we conducted a bootstrap analysis with a 95% bias-corrected confidence interval (CI) to assess the significance of indirect mediation-effects. If the CI does not include zero (0), it indicates that the indirect effect is statistically significant at a 0.05 level [72].

## 4. Results

### 4.1. Descriptive Statistics

Our research variables such as job insecurity, organizational identification and knowledge-hiding behavior were significantly related. The correlation-analysis results are shown in Table 2. 

### 4.2. Measurement Model

To test the discriminant validity of the main research-variables (job insecurity, coaching leadership, organizational identification, and knowledge-hiding behavior), we performed a CFA for all items, by checking the measurement model’s goodness-of-fit. To be specific, we compared our hypothesized four-factor model (which consists of job insecurity, coaching leadership, organizational identification, and knowledge-hiding behavior) with alternative models such as three-, two-, and one-factor models, by conducting a series of chi-square difference tests. 

The hypothesized four-factor model has a good fit (χ2 (df = 59) = 105.071; CFI = 0.984; TLI = 0.979; RMSEA= 0.048). We conducted a series of chi-square difference tests, comparing the four-factor model to a three-factor (χ2 (df = 62) = 554.702; CFI = 0.833; TLI = 0.790; RMSEA = 0.152), two-factor (χ2 (df = 64) = 1460.902; CFI = 0.527; TLI = 0.424; RMSEA = 0.252), and one-factor model (χ2 (df = 65) = 1937.842; CFI = 0.366; TLI = 0.239; RMSEA = 0.289). The results of the chi-square difference tests showed that the four-factor model was the best, indicating that our four research variables have an appropriate degree of discriminant validity.

### 4.3. Structural Model

We built a moderated mediation model that includes both mediation and moderation structures in the job-insecurity–knowledge-hiding-behavior link. In the mediation structure, the job-insecurity–knowledge-hiding-behavior link is mediated by the degree of an employee’s organizational identification. In the moderation structure, coaching leadership functions as a buffering factor, positively moderating the impact of employee job-insecurity on organizational identification.

Next, in the moderation structure, we multiplied job insecurity and coaching leadership. Before making the interaction term between the independent variable and moderator, all research variables (i.e., job insecurity, coaching leadership, organizational identification, and knowledge-hiding behavior) were centered on their means to increase the validity of the moderation analysis, by diminishing the degree of multi-collinearity between variables and minimizing the loss of correlations.

To assess how serious the multicollinearity bias was, we measured the values of variance inflation factors (VIF) and tolerances. The VIF values for job insecurity and coaching leadership were 1.02 and 1.02, respectively. Moreover, the values of tolerance were 0.98 and 0.98, respectively [73]. The VIF values are <10 with the tolerance values >0.2, indicating that job insecurity and coaching leadership are relatively free from multi-collinearity. 

#### 4.3.1. Result of Mediation Analysis

To find the best mediation model, we compared a full mediation model to a partial mediation model by performing a chi-square difference test. The full mediation model is identical to the partial mediation model except for the direct path from job insecurity to knowledge-hiding behavior. The fit indices of both the full mediation model (χ2 = 171.477 (df = 75); CFI = 0.956; TLI = 0.939; and RMSEA = 0.061) and the partial mediation model (χ2 = 157.099 (df = 74); CFI = 0.962; TLI = 0.947; RMSEA = 0.057) were acceptable. However, the chi-square difference test between the models (Δχ2 [1] = 14.378, *p* < 0.01) demonstrated that the partial mediation model was superior.

**Figure 2:**
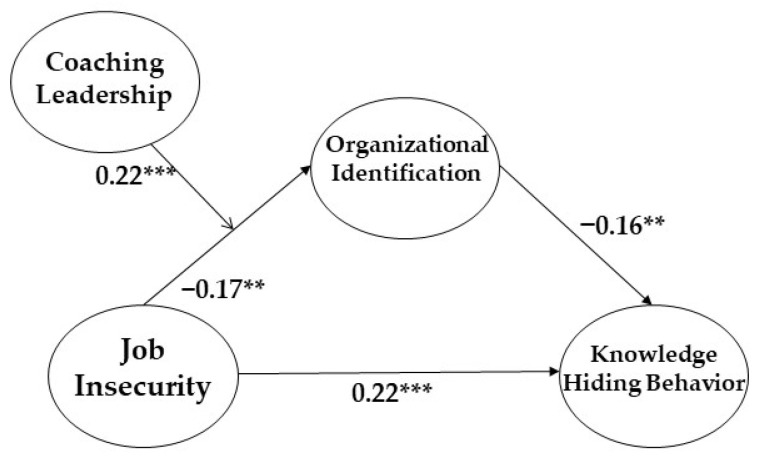
The Final Results of the Research Model. ** *p* < 0.01, *** *p* < 0.001.

Control variables such as gender, position, tenure, and education were not significant, except for gender (β = −0.13, *p* < 0.05). Including the control variables, our research model showed that job insecurity increased the degree of employee knowledge-hiding behavior (β = 0.22, *p* < 0.001), supporting Hypothesis 1, job insecurity decreased the degree of employee organizational-identification (β = −0.17, *p* < 0.01), supporting Hypothesis 2, and organizational identification also decreased the degree of knowledge-hiding behavior (β = −0.16, *p* < 0.01), supporting Hypothesis 3 (please see Figure 2).

#### 4.3.2. Result of Bootstrapping

To test whether organizational identification mediates the job-insecurity–knowledge-hiding-behavior link (Hypothesis 4), we conducted a bootstrap analysis with a sample of 10,000 [72]. The indirect mediation-effect would be significant at a 5% level if the 95% bias-corrected confidence interval (CI) for the effect of mean indirect-mediation excludes 0 [72]. 

The bias-corrected CI for the mean indirect-effect did not include 0 (95% CI = [0.005, 0.064]), which means that that the indirect sequential-mediation effect of organizational identification was statistically significant, supporting Hypothesis 4. The direct, indirect, and total effects of the paths from job insecurity to knowledge-hiding behavior are shown in Table 3.

#### 4.3.3. Result of Moderation Analysis

**Figure 3:**
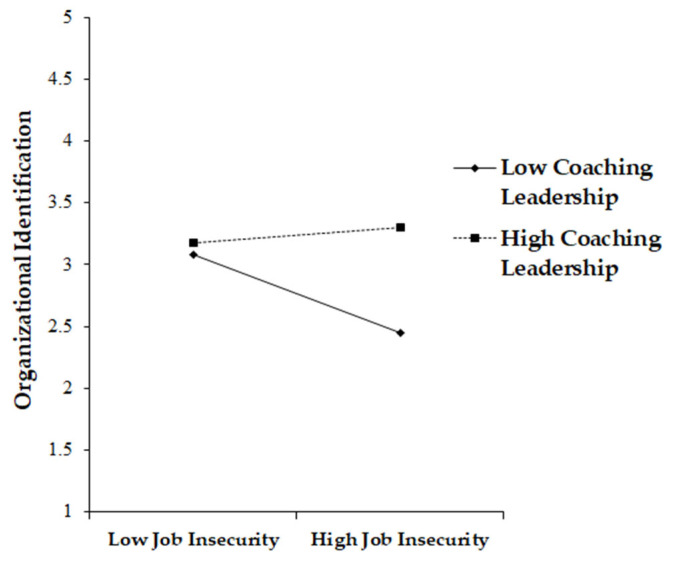
Moderating Effect of Coaching Leadership in the Job Insecurity–OI Link.

We tested the moderation effect of coaching leadership on the job-insecurity–organizational-identification link, by conducting a mean-centering process and making an interaction term. The coefficient of the interaction term (β = 0.22, *p* < 0.001) was statistically significant, which means that coaching leadership positively moderates the relationship between job insecurity and organizational identification, by playing a buffering role. When the level of coaching leadership is high, the decreasing impact of job insecurity on organizational identification can be diminished, supporting Hypothesis 5 (Please See Figure 3).

## 5. Discussion

We examined and tested the buffering role of contextual resources (coaching leadership) on the negative relationship between job insecurity and organizational identification. Using three-wave time-lagged cohort-study data from 346 Korean workers, we empirically found that employees who perceive job insecurity are less likely to feel organizational identification, leading to increased knowledge-hiding behavior. We also examined whether coaching leadership operates as a boundary condition in the negative relationship between job insecurity and organizational identification. We believe that the nested-data structure of this paper is less likely to influence our results, because each employee was matched with his or her own supervisor and organization. In other words, each employee has a different supervisor and organization. Thus, this research structure could make the data minimally interrelated. In addition, the research company randomly selected the participants. These are the reasons why we suggest that the data structure may have a minimum influence on our results. The overall results are consistent with the previous works on job insecurity, coaching leadership, organizational identification, and knowledge-hiding behavior [74,75,76,77,78,79]. Based on our results, theoretical and practical implications can be drawn. 

### 5.1. Theoretical Implications

We tested a moderated mediation model to simultaneously examine the underlying mechanisms of organizational identification and the moderating role of coaching leadership in the relationship between job insecurity and knowledge-hiding behavior. Our research contributes to the extant research as follows: first, we examined the relationship between job insecurity and knowledge-related behaviors. The relationships between job insecurity and behavioral outcomes (e.g., innovative behavior, voice behavior, organizational citizenship behavior, safety behavior, and counterproductive work-behavior) are well-documented [12,13,14,15,16]. However, surprisingly, research on knowledge-related outcomes is limited. In that knowledge is the driving force of organizational innovation and ultimately determines competitive advantage and success, it is necessary to examine the relationship between job insecurity and knowledge-related behavior. Therefore, this study enriches the job-insecurity literature by examining knowledge-related behavior.

Second, despite existing research calling for the need to explore additional underlying mechanisms and boundary conditions in the relationship between job insecurity and knowledge-related behaviors, empirical research examining intervening processes and boundary conditions is limited. To better understand why and when the relationship between job insecurity and knowledge-related behaviors occurs, it is important to examine mediators and moderators in the relationship. By integrating a psychological contract breach perspective and social identity theory, in this study we examined organizational identification as a mediating mechanism and coaching leadership as a boundary condition. In doing so, this research extends studies of the job-insecurity–knowledge-hiding-behavior association by adding a substantive intermediating mechanism and boundary condition to interpret how job insecurity impacts knowledge-hiding behavior, and when the impact of job insecurity is minimized or strengthened. 

Third, extant research on job insecurity has confirmed that leadership plays a critical role in buffering the negative impact of job insecurity. However, most studies have focused at the individual level on variables such as self-esteem, internal locus of control, proactive personality, psychological capital, resilience, and emotional intelligence and or macro-level contextual moderators such as labor-market insecurity, social safety networks, and macro-economic conditions [11,12]. As previous studies noted [26,27,28], leadership is a critical factor in encouraging subordinates’ perceptions, attitudes, and behaviors towards the organization. Thus, coaching leadership functions as a pivotal contingent factor in the relationship between job insecurity and knowledge-hiding behavior, via organizational identification. Our moderated mediation model highlighted the essential role of coaching leadership when understanding the influence of job insecurity on knowledge-hiding behavior.

### 5.2. Practical Implications

The results of our study suggest some practical implications. First, they show that job insecurity has important implications for knowledge-hiding behavior. By performing SEM, we empirically found that job insecurity leads to increased knowledge-hiding behavior. Organizational managers should keep in mind the fact that job insecurity hinders the flow of knowledge among organizational members, because knowledge is important for the achievement of organizational success and competitive advantage. Thus, beyond the material or financial incentives for preventing knowledge-hiding behaviors, it is even more important to reduce the extent of job insecurity of employees. To do so, organizations should implement human-resource management practices such as mentoring programs, long-term contracting with employees, and evaluating performance fairly [28]. 

Second, we suggest that organizational identification mediates the relationship between job insecurity and knowledge-hiding behavior. Decreased organizational-identification can increase the influence of job insecurity on knowledge-hiding behaviors. Thus, implementing specific measures to fortify employees’ organizational identification should be a concern for managers. They could increase the firm’s reputation via firm activities, systems, or lectures, inspiring employees to identify themselves as an organizational member and forming positive organizational images in their minds. Therefore, managers should pay attention to not only decreasing job-insecurity, but also increasing organizational-identification.

Third, we propose that coaching leadership buffers the negative impact of job insecurity on organizational identification. In particular, in the recent rapidly changing business environment, it is necessary to guide, facilitate, and inspire employees through coaching leadership, to help solve and cope with difficulties within the organization, such as job insecurity. By encouraging leaders to engage in coaching behaviors via training systems and courses (e.g., emphasizing the importance of subordinate guidance, discovering subordinates’ potentials and growth, providing subordinates with the opportunities to maximize their abilities), coaching leadership can be developed. 

### 5.3. Limitations and Future Studies

This study has theoretical and practical implications, but there are several limitations. First, the current paper should reconsider the choice of variables, to be specific, its mediator and moderator. We consider that the mediator (i.e., organizational identification), can be replaced by other similar and alternative concepts such as organizational commitment, perceived organizational support, and organization-based self-esteem (OBSE). In addition, strictly speaking, the target of knowledge-hiding behavior would be other employees rather than the organizations, although the employees are likely to perceive that their hiding behavior would be eventually harmful to the organization. Thus, future studies should investigate the influence of job insecurity on knowledge-sharing behavior instead of knowledge-hiding behavior.

Second, from the fundamental point of view, it is not easy for a leader to directly and accurately detect whether and how the employees hide their knowledge in an organization, because the intention to hide knowledge is less likely to be revealed outside. Although not only is the hiding behavior a kind of ‘behavior’, which is exposed outward like other behaviors in an organization, but the immediate leader is also likely to detect the employee’s knowledge-hiding intention and behavior through interacting with the subordinate in a frequent manner [67,80], the future works should consider this issue by selecting more obvious knowledge-related behavior, such as knowledge-sharing behavior.

Third, we believe that our nested-data structure was less likely to influence our results, because each employee was matched with his or her ‘own’ supervisor and organization, thus having a different supervisor and organization. Although this research structure could make the data minimally interrelated, we could not check this with an elaborated empirical method. Future studies should adequately deal with this issue. 

Fourth, we were only able to measure job insecurity with a subjective indicator, rather than with objective indicators such as unemployment or involuntary-turnover rates. Fifth, since this study was conducted among Korean employees, there is a limit to the generalization of our results. Therefore, future studies need to capture the impact of job insecurity using samples across other countries. Lastly, further research should examine other negative organizational-variables such as counterproductive work-behavior, deviance, and turnover intention, beyond the knowledge-hiding behavior used in this study. 

## 6. Conclusions

Relying on a psychological contract-breach-perspective, we delved into the influence of job insecurity on employee’s knowledge-hiding behavior. Our results demonstrated that job insecurity increased the degree of employees’ knowledge-hiding behavior via diminishing the degree of organizational identification. Moreover, coaching leadership functioned as a buffering factor that moderates the job-insecurity–organizational-identification link. This indicates that an employee’s organizational identification is an intermediating mechanism in the relationship between job insecurity and knowledge-hiding behavior. In addition, coaching leaders can buffer the negative influence of job insecurity. Although we acknowledge that this research has various limitations, we believe that this work can contribute to enhancing the job-insecurity and knowledge-hiding-behavior literature, through unveiling the underlying process and its contextual factor in the link.

## Figures and Tables

**Table 1:** Descriptive characteristics of the sample.

Characteristic	Percent
**Gender**	
Male	50.0%
Female	50.0%
**Age (years)**	
20–29	15.3%
30–39	35.8%
40–49	32.7%
50–59	16.2%
**Education**	
Below high school	8.7%
Community college	18.8%
Bachelor’s degree	61.6%
Graduate degree	10.9%
**Position**	
Staff	24.3%
Assistant manager	22.3%
Manager	22.5%
Deputy general manager	9.8%
Department/general manager	13.6%
Others	7.5%
**Tenure (** **years)**	
**Under 5**	48.3%
5 to 10	25.7%
11 to 15	13.9%
16 to 20	6.6%
21 to 25	2.0%
Above 26	3.5%
**Occupation**	
Office worker	71.7%
Profession (Practitioner)	7.8%
Manufacturing	6.1%
Public official	5.2%
Sales and marketing	4.6%
Administrative positions	2.9%
Education	0.3%
Others	1.4%
**Industry type**	
Manufacturing	24.6%
Wholesale/Retail business	12.4%
Construction	12.2%
Health and welfare	9.2%
Information service and telecommunications	8.7%
Education	8.1%
Services	6.4%
Financial/insurance	3.5%
Consulting and advertising Others	1.4%
Others	13.5%

**Table 2:** Descriptive statistics.

	1	2	3	4	5	6	7
1. Gender_T2	-						
2. Education	−0.12 *	-					
3. Tenure_T2	−0.28 **	0.04	-				
4. Position_T2	−0.40 **	0.20 **	0.34 **	-			
5. Job Insecurity_T1	−0.05	−0.02	−0.02	0.17 *	-		
6. Coaching Leadership_T1	−0.08	0.09	0.03	0.10	−0.14 **	-	
7. OI_T2	−0.17 **	0.10	0.17 **	0.19 **	−0.18 **	0.35 **	-
8. KHB_T3	−0.12 **	−0.08	0.09	0.06	0.24 **	−0.16 **	−0.17 **

Note: * *p* < 0.05. ** *p* < 0.01. S.D means standard deviation. OI means organizational identification, and KHB means knowledge-hiding behavior.

**Table 3:** Direct, Indirect, and Total Effects of the Final Research Model.

Model	Direct Effects	Indirect Effects	Total Effects
Job Insecurity -> Organizational Identificaiton -> Knowledge-Hiding Behavior	0.222	−0.026	0.248

All values are standardized.

## Data Availability

No new data were created or analyzed in this study. Data sharing is not applicable to this article.
